# Book and Pamphlet Notices

**Published:** 1858-07

**Authors:** 


### BOOK AND PAMPHLET NOTICES.

### REVIEW OF TULLY'S MATERIA MEDICA.

BY IRA HATCH, M. D., CHICAGO, ILL.

The first volume of Dr. Tully's great work on the Materia Medica is now complete and ready for sale. It is the work of a lifetime. It is the result of more than forty years of unwearied study and investigation, aided by great practical experience, by one of the first scholars of the age. No work that has ever preceded it combines so much originality, so much careful observation, and so profound research, as this work of Dr. Tully. His observations upon the *modus operandi* of medicines are worth all the work costs. The majority of the profession will undoubtedly dissent from his views at first; but the more they read and ponder, and compare their own experience and observations, the sooner they will adopt his views, to a great extent. His classification of medicines is founded upon the operative effects of each article, and its peculiarities in comparison with other articles, and also what it has in common with other articles.

This is a classification entirely new to the profession, and involving as it does the necessity of new names, will be distasteful to all those physicians who are opposed to study and investigation. A knowledge founded upon the medicinal powers, operations, or effects of remedies, is true science, and is almost the only scientific part of pure materia medica. The rejection of this sort of knowledge would reduce the department to the most complete empiricism. Every medicinal agent possesses certain specific powers, and produces certain effects or operations upon the human system. Every medicine has, also, different grades of operation. These different operations or effects, and different grades of operation or effects, are accurately defined and minutely described in the proems to his different classes of medicines. Nothing can be more indefinite and confused than the classification employed by most writers on the materia medica. Take, for instance, the class of medicines usually called stimulants. Under this head, medicines possessing effects as diverse as can well be conceived, are grouped together under the term stimulant, with a definition so comprehensive and unlimited that it actually has no meaning at all. Those medicines which are mere irritants are almost universally called stimulant, yet nothing is more unlike than irritation and stimulation. Exhilarants are always called stimulants, while it is well known to every practitioner who has watched their effect, that many of them at least had a depressing and exhausting effect, and are dangerous in all true atonic diseases. The same may be said of many other medicines, all of which ought to have distinct definitions under an appropriate class. Such a classification necessarily required new terms. These terms are well chosen. They are all derived from the Greek language, as most of the terms heretofore used have been. Dr. Tully has strictly adhered to this rule, and rejected some terms now in use, which are formed, contrary to all rules, from Greek and Latin. The term, *antacid*, composed of *anti*, Greek, and *acida*, Latin, is one of the old terms rejected.

In his *nomenclature*, he has also made extensive alterations. These alterations, though demanded by the progress of science, will probably meet with opposition by many of the profession who are attached to old names and old associations, and by others they will be met by ridicule, as savoring too much of pedantry and technicality. But having derived his terms from natural history and chemistry in every instance, the improvements which have been made in these sciences render a change of terms necessary in materia medica, in conformity with the two sciences from which it is derived.

On this subject, I cannot do better than to let the Doctor speak for himself. After quoting extensively from the most celebrated authors on this subject both in Europe and America, he says: "I shall therefore consider myself abundantly justified in introducing into the materia medica any of the changes in nomenclature which the progress of natural history and chemistry have made necessary in those sciences, since I should esteem it as almost criminal to deprive the materia medica of any improvements which it can derive from two branches with which it is so nearly connected, and upon which it so much depends. Chemistry and natural history are the two eyes of the materia medica.

"The nomenclature of natural history ought always to be the nomenclature of what are called the simples of the materia medica, while the nomenclature of chemistry ought always to be that of all chemical compounds, and such will be the fact in the succeeding work. From the earliest record of these several branches, such has always been the fact, and must ever continue to be the fact. But in modern times, in these departments of knowledge, it has been an objection to this community of terms, that improvements in natural history and chemistry (by which I understand the correction of errors and the addition of new facts) are constantly requiring improvements in terms, which seems to be considered an evil not to be endured in medicine. Although these objectors do not say so directly, they nevertheless seem to suppose that medicine is perfect, and ought now to remain forever unchanged and unchangeable. They would have what they call an *officinal nomenclature*." . . . . .

"There is always a great clamor whenever a writer changes a long employed term, or introduces a new term without disturbing an old one; and yet terms have been constantly undergoing change ever since human knowledge has been systematised and submitted to writing, and they doubtless will continue to be changed as long as knowledge continues to be progressive and is cultivated by the human race. Those who object to changes in chemical nomenclature, do not seem to be aware that they are, in fact, objecting to the progress of science; but this is actually the case. Under our present system, as I have already said, the name should exactly express the composition of the article, so that knowing the composition, may at once express the name, and knowing the name, may exactly indicate the composition.

"Now I presume that no one will presume to advocate the suspension of scientific progress in the discovery of the composition of substances, or the correction of any errors in relation to this point, into which we may have fallen, from imperfection of knowledge. But whenever we correct an error in composition, we must correct the name also. The *chlorides of mercury* will very fully illustrate this statement."

The changes which these preparations of mercury have undergone within a few years, in name, are too familiar to need any further illustration, and the reason is also known.

Whatever may be thought of Tully's materia medica, it may be studied with more profit than any other work on medicine that has appeared for the last fifty years. Its tendency will be to excite thought and investigation; to make its readers observe more closely and more accurately, and to awaken a deeper interest in the profession. The book will be worth all it will cost, for its correction of the many errors that prevail at present; for the example it sets of right reasoning in medicine, and of accuracy and precision in the use of medical language, to say nothing of its valuable principles of practice it inculcates, not found in any other work on the materia medica.

Dr. Bronson, of New Haven, Con., says: "It is the most important work on the subject of which it treats of the present century." The publisher, Dr. Church, has received the most flattering testimonials from the leading physicians in most of the large cities of this country and from several in Europe. He has lately had an order for the work from Vienna. The profession is much more largely indebted to Dr. Tully for his discoveries in medicine than most of them are aware. He has heretofore published but little from his immense pile of manuscripts, from which he has lectured in medical colleges for thirty years. But his students have pilfered from him, and published his teachings as their own original productions, as results of their own observations and experience. Dr. Norwood, of South Carolina, was a pupil of Dr. Tully, and to him he was indebted for his knowledge of veratrum viride. Many other articles of the materia medica have found their way into the profession in a similar manner. The second volume of the work now under consideration, which will treat of the different articles of the materia medica, is not yet published. The publisher has not the means for publishing it. If the first volume should not prove remunerative, the second may never appear. This event would be much regretted by all who are aware of the great amount of new and valuable knowledge, which would be lost to the profession.

The first volume is not so practical; it treats more of general principles; but the second volume will be wholly practical, and will contain a vast amount of valuable information which cannot be found in the books now in use.

Elements of Inorganic Chemistry, including the Applications of the Science in the Arts. By Thomas Graham, F. R. S. L. & E., late Professor of Chemistry in University College, London. Edited by Henry Watts, B. A., F. C. S., and Robt. Bridges, M. D. Second American, from the second and enlarged London edition. Complete in one volume, with two hundred and thirty-three illustrations on wood. Philadelphia: Blanchard & Lea, 1858. Pages 852. For sale by Keen & Co. of this city.

Graham's chemistry is too well and widely known to require anything more than the announcement of a new edition. This is an enlarged and improved issue of that familiarly known and deservedly popular work, brought up to the times, in a thorough and complete manner. It is now perhaps the best text book on the elements of inorganic chemistry extant, in any language or country.

Plates Illustrative of Wilson on Diseases of the Skin. Fourth Edition. Philadelphia: Blanchard & Lea, 1857.

We well remember the time when plates were thought gaudy luxuries in the study of our science, fitted only to sickly minds, and rather encouraging a mushroom growth of professional men, than as useful means of teaching. It was considered by the more favored student, that the substance instead of the shadow should be recognized as the only legitimate demonstrative means; in other words, that a man should always see every disease in hospital wards, and learn how to recognize and treat it there, before he could be trusted with the health and life of patients. A more careful consideration of the matter, however, ought to have taught the profession long ago, that plates not only served to freshen a retinal impression once experienced at the bedside, that certain external and non-fatal maladies might be demonstrated by them, almost as well as in any other way, to the saving of much valuable time.

Notwithstanding our former tardiness of appreciation of

"pictures," plates and painted ones are now regarded very properly as valuable in all departments of medical teaching. In fact, the rule now is to illustrate all our books in this way. This is right, and it would be right if we had ten windows to the soul instead of our five, to address them all; for by crowding in knowledge at every pore in the whole periphery of our being, we would not be able to imbue ourselves with all the knowledge of our profession. We believe the means, media and time are all that are requisite for us to attain to the acquisition of the whole mass of human knowledge, increasing as rapidly as it is, and there would be no danger either of our bursting or going mad, if it was all crowded in so small a space. Hence, we are glad to welcome any improvement that affords an additional medium for conveying knowledge to the mind. Among the many valuable books now being issued from the press, for the price and economy, this is the very best we know. It is a very complete set of illustrations of the diseases of the skin described in Wilson's excellent work on the subject, and should be in the hands of every private practitioner in America. Although diseases of the skin are less common in the country than in large cities, they are no less perplexing; in fact, their true character is less apt to be recognized. In this set of plates we have a key to the solution of all the cutaneous mysteries not otherwise attainable in many years of hard labor.

We have received also the Proceedings of the American Pharmaceutical Association, at the Sixth Annual Meeting, held in Philadelphia, September, 1857, with the Constitution and List of the Members. These proceedings are a worthy representation of this important branch of the science of medicine.

Also, Quarterly Summary of the Transactions of the College of Physicians of Philadelphia, from August 5th, 1857, to Feb. 3d, 1858, inclusive. This pamphlet contains many interesting items of intelligence, and evinces an active as well as capable condition of that Association.

Also, Fifteenth Annual Report of the Managers of the State

Lunatic Asylum of New York. Transmitted to the Senate, Feb. 7th, 1858. John P. Greary, M. D. is superintendent of this well established and useful institution, and in the discharge of his duties is doing honor to the profession he represents.

Prof. Alden March, of Albany, N. Y., has favored us with his production on the Intra-Capsular Fracture of the Cervix Femoris with Long Union, and an interesting case of Urinary Calculi, illustrated by engravings. In these illustrations and representations we have very strong proof of the bony union of the femoral neck within the capsular ligament. The work is characteristic by Dr. March's well known terseness, precision, and perspicuity.

### NEW JOURNALS.

OGLETHORPE MEDICAL AND SURGICAL JOURNAL. Edited by H. L. BYRD, A. M., M. D., Professor of the Principles and Practice of Medicine in Oglethorpe Medical College, and HOLMES STEELE, M. D., Professor of Obstetrics and Diseases of Women and Children in Oglethorpe Medical College. Nos. 1 and 2. Published monthly, $2.00 per annum. Savannah, Georgia.

Although young in years (or rather months), this offspring of medical literature shows considerable vigor and vitality, which promises well both for longevity and labor in the good cause. We extend the right hand of fellowship to Brothers Byrd and Steele, and wish them well in this new enterprise.

THE MAINE MEDICAL AND SURGICAL REPORTER. Conducted by W. R. RICHARDSON, M. D., and R. W. CUMMING, M. D., Proprietors. No. 1. Published monthly, at $3.00 a year, Portland, Maine.

Judging from the present number, we predict success in the editorial and mechanical concerns of the *Reporter*. And we hope and believe that with such intelligent management, pecuniary success will also attend the undertaking. We have placed it on our exchange list, and wish God-speed to our friends, Richardson and Cumming.

We should have noticed the change of place in the *Medical and Surgical Reporter*, formerly published at Burlington, New Jersey. Dr. S. W. Butler, the former sole, and now senior editor, has associated with him Dr. W. B. Atkinson, of Philadelphia, and proposes issuing that able periodical at Philadelphia in future. We hope this transplanting process may add new vigor and value to the *Reporter*. The soil and climate, judging from their productions for very many years, are certainly very favorable. Dr. Atkinson will doubtless be an addition of no small importance in conducting it.

